# Supplementary material for: Efficient Inhibition of Aspergillus flavus to Reduce Aflatoxin Contamination on Peanuts over Ag-Loaded Titanium Dioxide
Source: Toxins (Basel). 2023 Mar 10;15(3):216. doi: 10.3390/toxins15030216 (PMC10059131; doi:10.3390/toxins15030216)
Supplement: Supplementary file 1 [file toxins-15-00216-s001.zip › toxins-2256303-supplementary.pdf]

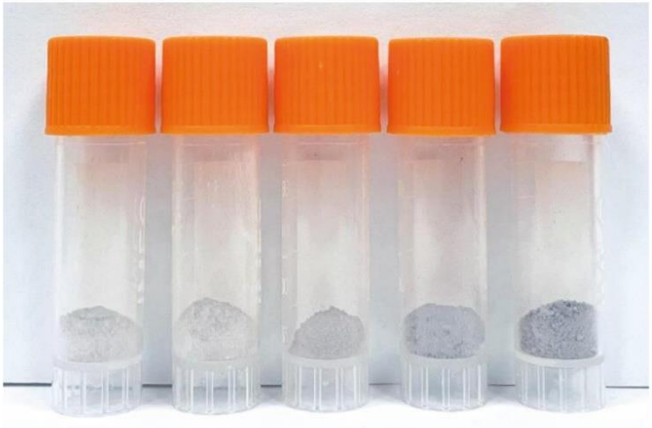

Figure S1. Ag/TiO_2 composites with silver concentrations of 0.5%, 1%, 1.5%, 2%, and 2.5%.

The Detailed Detection Method of Acid Value, Peroxide Value, Fat, Protein, Polyphenols, and Resveratrol

Acid Value

The acid value of peanuts was determined by the cold solvent indicator titration method of China National Standard, GB 5009.229-2016 [49]. Firstly, the peanuts were squeezed and centrifuged to obtain peanut oil. Then, 20 g of peanut oil were weighed and recorded it m. Then, 100 mL of ether-isopropanol (1:1) mixture and four drops of phenolphthalein indicator were added, and the mixture was shaken fully to dissolve the sample. Then, the samples were titrated with 0.1 mol/L potassium hydroxide standard titration solution. Titration was stopped immediately when the solution appeared reddish and had no obvious discoloration within 15 s, and the volume of the standard titration solution, used as V, was recorded. The volume used in the blank experiment was recorded as V_0. The acid value was calculated by the formula: $X_{AV} = (V - V_0) \times 0.1 \times 56.1/m$.

Peroxide Value

The determination of peanut peroxide value was adopted from the titration method by China National Standard, GB 5009.227-2016 [50]. The peanuts were squeezed and centrifuged to obtain peanut oil. Then, 3g of peanut oil (marked as m) were weighed into an iodine volumetric flask, 30 mL of chloroform-glacial acetic acid (4:6) mixture was added, and the mixture was shaken gently to dissolve the sample. Then, 1mL saturated potassium iodide solution was added, shaken evenly, and placed in the dark for 3min. 100 mL water was added, shaken again, and titrated with 0.002 mol/L sodium thiosulfate standard solution immediately. 1 mL of starch indicator was added when the solution turned light yellow appeared, and then we continued to titrate it until the blue disappeared in the solution. At the same time, a blank experiment was carried out. The volume of standard titrant consumed was marked as V and V_0 in the experimental group and blank group, respectively. The acid value was calculated by the formula: $X_{POV} = (V - V_0) \times 0.002 \times 0.1269 \times 100/m$.

Fat Content

According to the China National Standard, GB 5009.6-2016 [51], the Soxhlet extraction method was used to determine the fat content in peanuts. 2 g of ground peanuts were weighed (marked as m), and the mass of the receiving bottle was recorded as m_1. The fat in the peanut was extracted with anhydrous ether and then dried to constant weight at 105 °C. After cooling, the mixed weight of the receiving bottle and the fat were weighed as m_2. The crude fat content was calculated by the formula: $X = (m_2 - m_1)/m \times 100$.

Protein Content

According to the China National Standard, GB 5009.5-2016 [52], the Kjeldahl method was modified to determine the protein content in peanuts. Firstly, a 0.3 g peanut sample was weighed (marked as m) and placed in a digestive tube. Then, 10 mL of sulfuric acid and a mixed tablet containing copper sulfate and potassium sulfate were added into the digestive tube, which was subsequently placed in a digestion furnace and digested at 420 °C until the liquid was green and transparent. The blank experiment and sample experiment were carried out simultaneously. After cooling, the liquid was placed in a K9860 automatic Kjeldahl nitrogen analyzer (Shandong Haineng Future Technology Group Co., Ltd., sodium hydroxide solution, standard hydrochloric acid solution, and the boric acid solution containing mixed indicator were added before use) to determine the protein content. Protein content % = $(V_1 - V_2) \times c \times 1.4 \times 5.46 \times 100/(m \times V_3)$, where V_1 and V_2 were the volumes of standard titrant of hydrochloric acid consumed by the test solution and the blank group, respectively; c was the concentration of the standard titration solution of hydrochloric acid; and V_3 was the volume of the digestion solution.

Polyphenols content

The determination of polyphenol content in peanuts was carried out by modified Folin-Ciocalteu spectrophotometry (ISO 14502-1: 2005) [53]. We weighed 0.3 g of ground peanut samples and put them in 15 ml methanol solution (70%) to extract for 60 minutes. The supernate was centrifuged and taken out. Amounts of 5, 10, 20, 30, and 40 μg/mL gallic acid standard solutions were prepared at the same time. Folin-Ciocalteu, sodium carbonate solution, and H_2O were added to the above solution. The absorbance was measured with an Unicam UV 500 (Thermo, Waltham, MA, USA) at the wavelength of 765 nm. A standard curve was established with the gallic acid standard solution. The polyphenol content was calculated according to the standard curve.

Resveratrol Content

According to the China National Standard, GB 24903-2010 [54], the content of resveratrol in peanuts was determined by high-performance liquid chromatography (HPLC). An amount of 5 g ground peanut sample was extracted with 60 mL ethanol solution (85%) at 80 °C for 45 min in a water bath. The mixture was filtered and washed after cooling. All the filtrates were mixed, and then the solution volume was adjusted to 100 mL. An amount of 2 mL filtrate was taken, centrifuged at 5000 rpm/min for 5 min, and measured by high-performance liquid chromatography.
